# State Requirements and Recommendations for School-Based Screenings for Body Mass Index or Body Composition, 2010

**Published:** 2011-08-15

**Authors:** Jennifer Linchey, Kristine A. Madsen

**Affiliations:** University of California, San Francisco, Department of Pediatrics, San Francisco, California; University of California, San Francisco, Department of Pediatrics

## Abstract

**Introduction:**

We present a comprehensive picture of state requirements and recommendations for body mass index (BMI) and body composition screening of children and explore the association between pediatric obesity prevalence and state screening policies.

**Methods:**

Researchers completed telephone interviews with contacts at the departments of education for all 50 states and reviewed state content standards for physical education.

**Results:**

Twenty states (40%) require BMI or body composition screening, and 9 states (18%) recommend BMI screening or a formal fitness assessment that includes a body composition component. The prevalence of adolescent obesity was higher in states that require BMI screening or fitness assessments with body composition than in states without requirements (16.7% vs 13.6%, *P* = .001).

**Conclusion:**

Future studies should evaluate the effect and cost-effectiveness of BMI and body composition screening on child obesity.

## Introduction

Pediatric obesity remains an important public health concern (1-3). School-based body mass index (BMI) screening has been recommended to address pediatric obesity (4), and recent reports suggest that approximately 25% of states require BMI measurement in schools (5,6). States may also be screening for BMI or, more generally, body composition through comprehensive fitness assessments. Many national fitness assessments include a measure of body composition such as BMI or skinfold measurements to assess students' weight-related cardiovascular risk (7-9). The Institute of Medicine suggests that body composition screenings can increase parents' awareness of their child's weight-related risks, which may lead to lifestyle changes and improvements in pediatric obesity (4).

Although recent reports have identified states that require BMI screening (5,6), data are not available on states that recommend BMI screening or require BMI screening as part of a comprehensive fitness assessment. Thus, existing reports on BMI and fitness screening likely underestimate the extent of and number of students exposed to such screening. Further, not all fitness assessments include a measure of body composition, and available reports do not indicate which states' fitness assessments include BMI or body composition. To explore the effect of school-based BMI and body composition screening on obesity, it would be useful to identify states that require or recommend such screening, either alone or through comprehensive fitness assessments, and to determine whether obesity prevalence at the state level is associated with screening practices. Knowing states' varying policies related to BMI and body composition assessment would also support comparisons of the effects of different screening approaches. In this study, we sought to present a comprehensive picture of state requirements and recommendations for BMI and body composition screening and to determine whether pediatric obesity prevalence is associated with state screening policies.

## Methods

### Telephone interviews

In the fall of 2010, researchers called the main telephone line for the department of education for each of the 50 states and asked to speak with the person most knowledgeable about school-based health screenings. The appointed person was asked to confirm that he or she was the most appropriate contact with whom to speak. All interviews occurred at the time of contact and typically lasted 10 minutes. Contacts (84%) predominantly held the titles of coordinator (24%), consultant (26%), program specialist (22%), or director (12%), and most worked within the divisions of curriculum and instruction or school health.

Using a structured telephone interview script, researchers asked respondents whether their state had a requirement or recommendation for school-based BMI measurement or fitness assessment. When state contacts reported that their state required fitness assessments, researchers asked whether the fitness assessment included BMI or another body composition component. Researchers elicited assessment protocols (frequency of collection, participating grade levels, use of nationally recognized fitness assessment tools, and policies on parent notification) from contacts who reported a state requirement or recommendation for either BMI measurement or body composition assessment via a complete fitness test.

Researchers verified responses from department of education contacts against the *Shape of the Nation* report published in 2010 (6), which contains information on states' required school-based BMI and fitness assessment policies. If requirements as stated in telephone interviews differed from the *Shape of the Nation* report, researchers called the state contact listed in the *Shape of the Nation* report to resolve the discrepancy.

### State content standard for physical education

According to the American Alliance for Health, Physical Education, Recreation and Dance, content standards for physical education (PE) provide "the framework for a quality physical education" (10). Researchers reviewed the content standards for PE (6) for each state in which contacts did not report a state requirement or recommendation for BMI screening or fitness assessment. When language in PE content standards promoted use of a nationally recognized fitness assessment tool that included body composition, researchers documented the suggested assessment tools and grade levels.

### Fitness assessment tools

Fitness assessment tools used for school-based screenings include Adapted Physical Education Assessment Scale (APEAS) (7), Brockport Physical Fitness Test (Brockport) (8), Fitnessgram (9), and the President's Challenge (11). APEAS consists of up to 23 items that assess motor control, physical fitness, and BMI. Brockport offers 27 tests to measure muscular strength and endurance, flexibility, aerobic capacity, and body composition. Brockport lists both BMI and skinfolds as options for measuring body composition. Fitnessgram similarly assesses muscular strength and endurance, flexibility, aerobic capacity, and body composition, recommending use of skinfolds but listing BMI and bioelectrical impedance as alternate measures of body composition. Bioelectrical impedance, which determines the body's impedance (ie, opposition) to a small electrical current, allows the estimation of the fat-free body mass and percent body fat. In California, which requires annual Fitnessgram assessments, approximately 95% of school districts measure BMI to fulfill the body composition requirement (Julie Williams, California Department of Education, written communication, April 2010). The President's Challenge comprises 5 tests that measure muscular strength and endurance, aerobic capacity, speed, agility, and flexibility, but do not assess body composition.

### State statistics

Researchers obtained data on prevalence of obesity (BMI ≥95th percentile for age and sex) by state for children aged 10 to 17 years from the 2007 National Survey of Children's Health (NSCH) (12). The 2007 NSCH was a random-digit–dialed telephone survey conducted from April 2007 through July 2008 with a sample size of approximately 1,800 children per state from birth to age 17 years (13). In the 2007 NSCH, only parents of children aged 10 to 17 years were asked to provide their child's height and weight.

Researchers obtained data on public school enrollment for grades 1 through 12 in the 2008-2009 school year from the US Department of Education's National Center for Education Statistics (14). We used the Wilcoxon rank sum test to compare public school enrollment and linear regression to compare prevalence of obesity (adjusting for enrollment) between states with and without screening policies. Calculations were performed by using Stata/MP 11.1 for Mac (StataCorp LP, College Station, Texas).

## Results

Telephone interviews revealed that 20 (40%) states require BMI or body composition screenings; 13 states require BMI screening, and 7 states require a complete fitness assessment that includes BMI or body composition (Table 1). Of these 20 states, 4 require and 3 recommend use of Fitnessgram; no other assessment tools were mentioned. Alabama required the President's Challenge through the 2009-2010 school year; a new state-developed assessment tool that includes BMI was piloted during 2010-2011 and will be implemented beginning in 2011-2012. Nine of the 20 states requiring BMI or body composition screening also require parent notification (Table 1), and all states require assessments at least annually. In 2 states, contacts did not report that BMI screening was required or recommended, but the *Shape of the Nation* report suggested that BMI screening was required. Telephone calls with the contact in the *Shape of the Nation* report (which was the initial telephone interviewee in 1 case) revealed that in both states, height and weight assessments were required but BMI was not calculated (Table 1). Three additional states (Delaware, Missouri, and Virginia) require complete fitness assessments but have opted to make body composition measurement optional.

Among states not requiring screening, New Hampshire recommends BMI screening and Hawaii recommends the use of a fitness assessment that includes body composition. The PE content standards of an additional 7 states promote use of a fitness assessment that includes body composition. Hawaii and the additional 7 states that include body composition in their content standards all suggest use of Fitnessgram, 2 also suggest use of Brockport, and 1 suggests use of APEAS as an option (Table 2).

Compared with states without requirements, the 20 states that require BMI screening or fitness assessments with body composition were more populous (median public school enrollment 900,000 vs 500,000 students, *P* = .007) and were 3.1 percentage points higher in adolescent obesity (16.7% vs 13.6%, *P* = .001), adjusting for public school enrollment. The 7 states requiring BMI as part of a fitness assessment were 4.9% percentage points higher in obesity than states that did not require assessment (*P* = .001), and the 13 states requiring BMI screening alone were 1.7 percentage points higher in obesity than states that did not require assessment (*P* = .10). States recommending BMI or fitness assessment had no greater enrollment or rates of obesity than states neither requiring nor recommending screenings.

North Carolina and Maryland neither require nor recommend screening or fitness assessment, but state contacts reported that more than 75% of school districts statewide are using a fitness assessment that includes body composition. Contacts in 3 additional states, Oklahoma, Wisconsin, and Kansas, reported that they were conducting pilot studies of Fitnessgram and planned to increase use in coming years.

## Discussion

The study findings suggest that relying on reports of required school-based BMI screening greatly underestimates the number of students who have BMI or body composition screened. This study extends data in the *Shape of the Nation* report, which neither assessed recommendations for screening nor distinguished between fitness assessments that did and did not include body composition (6). According to the *Shape of the* *Nation* report, the percentage (26%) of states that specifically required BMI screening in 2010 increased only slightly from the 22% reported in the 2006 *School Health Policies and Programs Study* (SHPPS) (5,6). However, we found that an additional 14% of states require comprehensive fitness assessment that includes body composition, and 18% recommend BMI or fitness assessment; neither were collected in SHPPS. Thus, we found that BMI or body composition screening, either alone or as part of a comprehensive fitness assessment, was required by 20 states, representing nearly 30 million school-aged children (14), and was recommended by another 9 states, representing almost 4 million children.

Recommendations for screening likely helped to increase BMI screenings, as data from SHPPS indicated that only 22% of states required BMI screening but more than 40% of schools enacted the practice (6). Although the SHPPS data may in part reflect that states requiring screening are more populous than other states (as our data indicate), clearly screening also occurs voluntarily. In our study, contacts from 5 states without requirements or recommendations for screening reported widespread use or pilot testing of comprehensive fitness assessments that include body composition. Additionally, screening policies may not be enforced and, even in states with required screening, actual prevalence of screening is unknown. Studies that gather data at the school level will be informative, particularly studies with questions about comprehensive fitness assessments that include BMI or body composition screening as well as BMI screening in isolation.

To our knowledge, this is the first study to examine the relationship between state policies related to body composition screening and obesity prevalence. We found that the prevalence of obesity is higher in states that require screening than in those that do not. It is possible that states with the greatest prevalence of obesity have instituted screening policies to improve the health of their children, although this cross-sectional study cannot assess causality. Reporting results to parents might help improve child health, yet only 8 states require that parents be notified of screening results. Nonetheless, it is likely that parent reporting occurs frequently even when not required. In California, where annual body composition testing is required but reporting results to parents is optional, half of school districts elect to notify parents (15).

Given that states require or recommend Fitnessgram more than other assessment tools, future evaluations of body composition screening and reporting should include Fitnessgram. Specific attention should be given to reporting methods, as results from a study in California suggest that current methods of body composition reporting do not alter pediatric obesity at the population level (15). One area for research is comparing the effectiveness of reporting BMI versus percent body fat results to parents to promote family lifestyle changes. Although the Fitnessgram specifically recommends using skinfolds to assess body composition, in the nonresearch setting, skinfolds may be less accurate than height and weight and may not be better in predicting body fat than using a BMI cutoff at or above the 95th percentile (16). Whether BMI or skinfold assessments are used, future research should also compare the effect of reporting body composition alone versus multiple results from a comprehensive fitness screening on child health. In our study, states with the highest prevalence of obesity were more likely to require the comprehensive fitness assessments than BMI screening alone. Further, to assist states in resource allocation decisions related to child health, the equipment and software required for some comprehensive fitness assessments should be considered in cost-effectiveness analyses.

The 9 states that recommend BMI screening or fitness assessment offer an opportunity for research on the frequency of such assessments in the absence of a specific policy. Studying variation in the adoption of recommendations and adherence to PE content standards among states may shed light on how to most effectively implement policies addressing pediatric obesity.

This study had several limitations. Contacts may not have provided accurate information, and information at the state level may not reflect what happens locally. Thus, estimates of screening prevalence may be too high or too low. Information on obesity prevalence came from parents' reports, which may be inaccurate and subject to bias. However, even if estimates of obesity prevalence at the state level are biased, it is difficult to determine how this would affect the demonstrated associations between obesity prevalence and screening policies.

School-based BMI and body composition screening policies are widespread. Further studies examining the effect of school-based screening practices on obesity are needed. Our study, which documents the prevalence of BMI and body composition screening across the United States, will allow those researching obesity-related policy to target states at various stages of screening implementation.

## Figures and Tables

**Table 1:** BMI or Body Composition Assessment Protocols Among 20 States Requiring Screening in Schools, 2010

**13 States Requiring BMI Screening**			

**State**	**Tools Used to Assess BMI or Body Composition**	**Grade Levels**	**Parent Notification Required?**
Arkansas	None required or recommended	K, 2, 4, 6, 8, and 10	Yes
Connecticut^a^	None required or recommended	K, 6 or 7, and 9 or 10	No
Florida	None required or recommended	1, 3, 6, and optionally 9	No
Illinois^b^	None required or recommended	Before entering school and in grades K or 1, 6, and 9	Yes
Maine	None required or recommended	Rules have not yet been promulgated	
Massachusetts	None required or recommended	1, 4, 7, and 10	Yes
Nebraska^c^	None required or recommended	Preschool, K-4, 7, and 10	No
Nevada	None required or recommended	4, 7, and 10	No
New Jersey^a^	None required or recommended	1-12	No
New York^b^^,^ d	None required or recommended	Upon school entry and in grades pre-K or K, 2, 4, 7, and 10	Yes
Ohio	Fitnessgram^e^ recommended	K, 3, 5, and 9	Yes
Pennsylvania	None required or recommended	1-12	Yes
Tennessee^d^	Fitnessgram^e^ recommended	K, 2, 4, 6, 8, and 1 year of high school	Yes

**7 States Requiring Body Composition Assessment as Part of a Complete Fitness Assessment**			

Alabama	Alabama Physical Fitness Assessment required starting 2011	2-8 and high school students enrolled in PE	Yes
California	Fitnessgram^e^ required	5, 7, and 9	No
Georgia^f^	Fitnessgram^e^ required	1-12 (students enrolled in PE)	Yes
Mississippi	None required or recommended	5 and high school students enrolled in PE	No
South Carolina	Fitnessgram^e^ recommended	5, 8, and high school students enrolled in PE	No
Texas	Fitnessgram^e^ required	3-12	No
West Virginia	Fitnessgram^e^ required	4-8 and 1 year of high school	No

Abbreviations: BMI, body mass index; K, kindergarten; PE, physical education.

a Height and weight measurements are required but calculation of student BMI is not.

b Students may opt to bring BMI measurement from a medical provider.

c BMI screening will be required starting in the 2011-2012 school year.

d State requires parent notification when BMI poses a health concern on the basis of state-defined criteria.

e Fitnessgram assesses body composition.

f Fitness assessment piloted in 2010-2011; will be mandated starting 2011-2012 school year.

**Table 2:** States That Recommend Assessing BMI or Body Composition Either Through Screening or Inclusion of Fitness Assessments in Physical Education Content Standards, 2010

**State**	**Content Standards**	**Tools Used to Assess BMI or Body Composition**	**Grade Levels**
Alaska	Alaska Physical Education Standards	Fitnessgram, Adaptive Physical Education Assessment, Brockport Physical Fitness Test	3-12
Hawaii	NA	Fitnessgram	Not specified
Idaho	Idaho Content Standards for Physical Education	Fitnessgram	3-high school
Louisiana	Louisiana Physical Education Content Standards Bulletin 102	Fitnessgram	4-high school
Michigan^a^	Physical Education Content Standards and Benchmarks	Fitnessgram, Brockport Physical Fitness Test	K-12
New Hampshire^b^	NA	None recommended	K-12
Rhode Island^a^	The Rhode Island Physical Education Framework	Fitnessgram	2-12
Utah	Utah State Office of Education Physical Education Core Curriculum	Fitnessgram	3-6
Vermont	Grade Expectations for Vermont's Framework of Standards and Learning Opportunities — Physical Education	Fitnessgram	3-high school

Abbreviations: BMI, body mass index; NA, not applicable; K, kindergarten.

a State also lists President's Challenge as an option for fitness assessment. President's Challenge does not include a body composition component.

b New Hampshire specifically recommends BMI screening, which does not occur as part of a complete fitness assessment.

## References

[1] Daniels SR, Arnett DK, Eckel RH, Gidding SS, Hayman LL, Kumanyika S (2005). Overweight in children and adolescents: pathophysiology, consequences, prevention, and treatment. Circulation.

[2] Freedman DS, Mei Z, Srinivasan SR, Berenson GS, Dietz WH (2007). Cardiovascular risk factors and excess adiposity among overweight children and adolescents: the Bogalusa Heart Study. J Pediatr.

[3] Ogden CL, Carroll MD, Curtin LR, Lamb MM, Flegal KM (2010). Prevalence of high body mass index in US children and adolescents, 2007-2008. JAMA.

[4] (2005). Preventing childhood obesity: health in the balance. Institute of Medicine, Committee on Prevention of Obesity in Children and Youth.

[5] Brener ND, Wheeler L, Wolfe LC, Vernon-Smiley M, Caldart-Olson L (2007). Health services: results from the School Health Policies and Programs Study 2006. J Sch Health.

[6] (2010). 2010 Shape of the nation report: status of physical education in the USA.

[7] Adapted Physical Education Assessment Scale. American Association for Physical Activity and Recreation, a national association of the American Alliance for Health, Physical Education, Recreation and Dance.

[8] Winnick JP, Short FX (1999). Brockport fitness test manual.

[9] Welk GJ, Meredith ME (2008). Fitnessgram/Activitygram reference guide.

[10] Moving into the future: National Standards for Physical Education.

[11] (2010). We are All-Americans: strong kids for a strong nation. The President's Challenge.

[12] National Survey of Children's Health. The Child and Adolescent Health Measurement Initiative.

[13] Blumberg SJ, Foster EB, Frasier AM, Satorius J, Skalland BJ, Nysse-Carris KL (2009). Design and operation of the National Survey of Children's Health, 2007. Vital and Health Statistics.

[14] 2008-09 State enrollments by grade. National Center for Education Statistics: Elementary/Secondary Information System.

[15] Madsen KA School-based BMI screening and parent notification: a statewide natural experiment. Arch Pediatr Adolesc Med.

[16] Himes JH (2009). Challenges of accurately measuring and using BMI and other indicators of obesity in children. Pediatrics.

